# Enhancing image security via chaotic maps, Fibonacci, Tribonacci transformations, and DWT diffusion: a robust data encryption approach

**DOI:** 10.1038/s41598-024-62260-3

**Published:** 2024-05-29

**Authors:** Mohammad Mazyad Hazzazi, Mujeeb Ur Rehman, Arslan Shafique, Amer Aljaedi, Zaid Bassfar, Aminu Bello Usman

**Affiliations:** 1 Department of Mathematics, College of Science, King Khalid University, 61413 Abha, Saudi Arabia; 2 Cyber Technology Institute, School of Computer Science and Informatics, De Montfort University, Leicester, LE1 9BH UK; 3 School of Electronic and Nanoscale Engineering, University of Glasgow, Glasgow, G12 8QQ UK; 4 College of Computing and Information Technology, University of Tabuk, Tabuk, 71491 Saudi Arabia; 5 College of Computing and Information Technology, University of Tabuk, Tabuk, 71491 Saudi Arabia; 6 School of Computer Science, University of Sunderland, Sunderland, SR1 3SD UK

**Keywords:** Engineering, Mathematics and computing

## Abstract

In recent years, numerous image encryption schemes have been developed that demonstrate different levels of effectiveness in terms of robust security and real-time applications. While a few of them outperform in terms of robust security, others perform well for real-time applications where less processing time is required. Balancing these two aspects poses a challenge, aiming to achieve efficient encryption without compromising security. To address this challenge, the proposed research presents a robust data security approach for encrypting grayscale images, comprising five key phases. The first and second phases of the proposed encryption framework are dedicated to the generation of secret keys and the confusion stage, respectively. While the level-1, level-2, and level-2 diffusions are performed in phases 3, 4, and 5, respectively, The proposed approach begins with secret key generation using chaotic maps for the initial pixel scrambling in the plaintext image, followed by employing the Fibonacci Transformation (FT) for an additional layer of pixel shuffling. To enhance security, Tribonacci Transformation (TT) creates level-1 diffusion in the permuted image. Level-2 diffusion is introduced to further strengthen the diffusion within the plaintext image, which is achieved by decomposing the diffused image into eight-bit planes and implementing XOR operations with corresponding bit planes that are extracted from the key image. After that, the discrete wavelet transform (DWT) is employed to develop secondary keys. The DWT frequency sub-band (high-frequency sub-band) is substituted using the substitution box process. This creates further diffusion (level 3 diffusion) to make it difficult for an attacker to recover the plaintext image from an encrypted image. Several statistical tests, including mean square error analysis, histogram variance analysis, entropy assessment, peak signal-to-noise ratio evaluation, correlation analysis, key space evaluation, and key sensitivity analysis, demonstrate the effectiveness of the proposed work. The proposed encryption framework achieves significant statistical values, with entropy, correlation, energy, and histogram variance values standing at 7.999, 0.0001, 0.0156, and 6458, respectively. These results contribute to its robustness against cyberattacks. Moreover, the processing time of the proposed encryption framework is less than one second, which makes it more suitable for real-world applications. A detailed comparative analysis with the existing methods based on chaos, DWT, Tribonacci transformation (TT), and Fibonacci transformation (FT) reveals that the proposed encryption scheme outperforms the existing ones.

## Introduction

With the exponential and, frequency use of wireless technology and digital devices, the exchange of digital data such as images has increased the demand for robust security^1,2^. As images play a vital role in communication, ensuring image security has become an important concern. Numerous approaches, such as secret image sharing^3^, image steganography^4^, and image encryption^5,6^, proposed in the past several years to enhance the security of image transmission through wireless channels such as the Internet. Digital images are characterized by their significant data content, which exhibits high pixel correlation and redundancy. Consequently, traditional cryptographic schemes like DES and AES are not suitable for image encryption due to their high computational complexity. To address this challenge, several categories of encryption schemes have been proposed to effectively protect the image data^7–11^.

The image encryption algorithm is used to convert an image into an unreadable message known as a cipher image through the utilization of secret keys^12^. This resulting cipher image bears a resemblance to a noisy image, rendering it virtually difficult for attackers to recover the plaintext image from the cipher image. This transformation is primarily achieved either by scrambling the pixels or by changing the intensity of the original pixel values. Specifically, the pixel scrambling technique plays a crucial role in breaking the correlation of the adjacent pixels. This phase, appropriately termed the confusion phase of image encryption, results in a cipher image with an entirely different appearance. In particular, this pixel scrambling process preserves the histogram of the original image since it does not alter the pixel intensity^13^. Subsequently, the diffusion phase emerges as an integral step in the image encryption process, aimed at modifying the intensity values of the pixels. This modification leads to the establishment of a uniform histogram in the cipher image, thereby enhancing the system’s resilience against a spectrum of potential attacks^14^. The detailed analysis of the gray-scale images including medical images can be found in^15^.

Image encryption plays a pivotal role in various essential functions within the corporate world^16^. In the realm of healthcare, the digitization of health records, which encompasses patient data, medical history, and symptoms, requires robust protection due to their sensitive nature^17^. Securing these records from unauthorized access is paramount. Likewise, in the military sector, images such as maps, building locations, and intelligence on adversaries are of the utmost importance. They play vital roles in tasks like small target identification, tracking, and missile guidance. Consequently, ensuring the security of these images is critical to national defense, as unauthorized access could pose significant threats. The media industry, characterized by round-the-clock news broadcasting, hinges on the privacy of multimedia content, be it images, audio, or video. Image encryption plays a crucial role in safeguarding this information from unwarranted intrusion. Additionally, as cloud storage becomes increasingly prevalent, third-party entities store client data in the cloud, including images. Protecting the privacy of these cloud-stored images is imperative to maintaining data security and confidentiality, making image encryption an essential component of cloud applications^18^.

**Figure 1:**
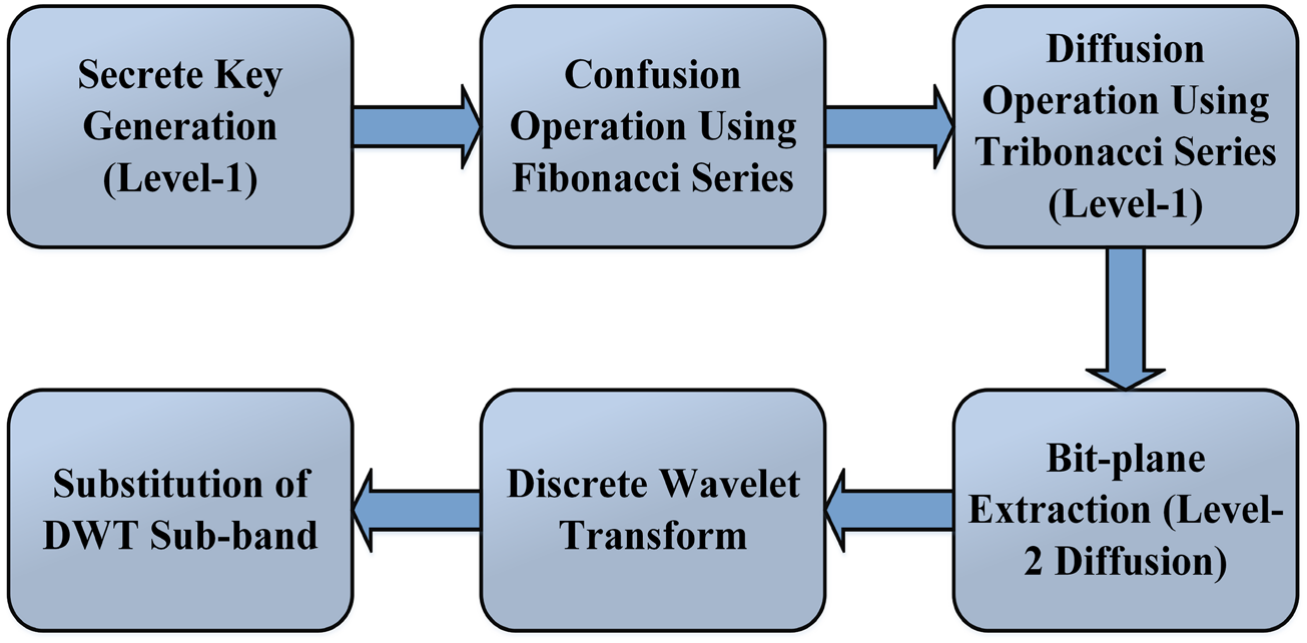
Generic flow of the proposed work.

The proposed research introduces a novel image encryption method that employs the Fibonacci transform (FT) to scramble pixels, achieved through matrix multiplication of two matrices ($2 \times 2$ and $2 \times 1$). The diffusion is created using the XOR operation in the scrambled image with a randomly generated i-key image. A key innovation lies in the utilization of the Tribonacci-based transformation (TBT) to modify pixel values, which is achieved by multiplying a $3 \times 3$ Tribonacci array with a $3 \times 1$ vector representing three consecutive pixels. Further in the diffusion process, traditional operations such as substitution box (S-box) and circular bit-shift are applied. Moreover, the proposed method demonstrates the ability to handle different categories of images, such as binary images and images with different levels of grayscale. It is lauded for its simplicity, ease of implementation, and fast execution. Experimental results show the resilience of the proposed method. The generic flow of the proposed work is displayed in Fig. 1.

The proposed research holds significant potential for various practical applications where image security is paramount. For instance, for sensitive data storage, secure communication channels, and confidential image sharing, the integration of chaotic maps, Fibonacci, Tribonacci transformations, and Discrete Wavelet Transform (DWT) diffusion provides a robust data encryption framework. The innovative fusion of these cryptographic techniques aims to provide robust security measures to ensure the protection of critical visual information. This research’s findings could find application in sectors ranging from medical imaging and military communications to secure digital information, which contributes to the securing of sensitive visual data in an increasingly interconnected and data-driven world.

### Motivation

In today’s digital age, the exchange and transmission of sensitive information, particularly through visual media like images, demands robust security measures. In the year 2020, Equifax, a leading credit reporting company, suffered a substantial data breach, leading to the compromise of private personal information, including images and credit card details, for 143 million individuals^19^. According to a study presented in^20^, around 52% of digital information in several countries such as Bangladesh, Nepal, Sweden, and Denmark is categorized as facing high cyber risk, while 32% is deemed to have a moderate risk, and a mere 12% is considered to be at low risk. The study shows the considerable cyber threat landscape confronting sensitive information, with an average of 630 cyberattacks directed at digital data such as images and bank credit cards. Therefore, with the increasing prevalence of cyber threats and unauthorized access, the need for effective cryptographic protocols has become paramount. As per cybersecurity reports, sensitive images often become targets for malicious activities due to their vulnerability during transmission. Image encryption, when implemented effectively, secures against unauthorized access and ensures the confidentiality and integrity of the visual data.

Considering these challenges and the evolving landscape of cyber threats, the proposed research aims to address the need for a robust ecryptographic protocol that provides a strong defense against cyberattacks while maintaining a low processing time. The proposed encryption framework combines chaotic maps, Fibonacci and Tribonacci transformations, and DWT diffusion to achieve an efficient solution for enhancing image security. By integrating these elements, the proposed approach aims to surpass existing methods in terms of security and real-time applicability, which contributes to the ongoing efforts to enhance digital data protection.

### Organization of the paper

The rest of the sections of this paper are structured as follows: "Related work" section provides an overview of existing encryption schemes, accompanied by a summary table outlining vulnerabilities and potential solutions. "Preliminaries" section gives the foundational knowledge essential for developing the proposed encryption scheme, detailed in "Proposed encryption procedure" section. The experimental outcomes of the proposed encryption framework are explained in "Experimental results and analysis" section. "Uniqueness and the advantages of the proposed encryption framework" section is devoted to outlining the advantages and uniqueness of the proposed work. It reveals the advantages and shows the uniqueness of the proposed encryption framework in comparison to existing studies. Finally, "Conclusion and future work" section concludes the proposed research and provides a few future recommendations aimed at enhancing the proposed encryption framework.

## Related work

In the early stages of image encryption development, the focus was primarily on applying these methods to compressed data. Among the various categories of image encryption techniques, chaotic-based approaches have garnered substantial attention^21^. Their popularity stems from their sensitivity to initial parameters, the capacity to generate pseudorandom sequences, and the unpredictability of motion patterns. These chaotic methods involve the computation of pseudo-random sequences, which are then used to define specific permutations for tasks like pixel scrambling, bit plane permutation, and substitution matrix creation^22–24^.

The chaotic behavior aligns well with the encryption requirements, improving security. Early researchers predominantly used classical chaotic maps such as Baker’s map^25^, logistic map^26^, and tent map^27^. Recently, innovative hybrid chaotic image encryption methods have emerged, combining chaotic maps with optimized substitution boxes^28–31^. Some of the recent advancements involve the adoption of high-dimensional chaotic systems^32–34^. These systems have gained popularity due to their ability to expand the key space and improve resilience against attacks. For instance, a 5D hyperchaotic system^35^ has been proposed to encrypt color images, involving the decomposition of plain images into sub-bands through complex wavelet transformations. Subsequently, secret keys derived from a 5D chaotic map are used to diffuse these sub-bands. Furthermore, novel visual encryption schemes featuring 6D hyperchaotic systems^36^ and image cryptosystems utilizing hyperchaotic systems in conjunction with Fibonacci Q-matrices have been introduced in recent research.

### Cahos based image encryption schemes

Encryption schemes for images based on chaos utilize the inherent unpredictability and sensitivity to initial conditions present in chaotic systems to safeguard digital images. For instance, in^37^, Nematzadeh et al proposed a new hybrid method based on the integration of DNA encoding and a Binary Search Tree (BST) algorithm. In^38^, Yadollahi et al. proposed a two-phase secure image encryption scheme using DNA and RNA. It generates the initial cipher image using DNA rules, and a chaotic function, while the final encrypted image is obtained through the use of XOR operation. In^39^, Abbasi et al. proposed a new Chaotic Evolutionary Biomolecules Model (CEBM) for image encryption, which incorporates the concepts of DNA and RNA-based encryption methods^40^. This model employs multiple chaotic maps for scrambling purposes, while DNA, RNA, and XOR operations are used to create the diffusion in the plaintext image^41–43^. In^28^, Benaissi et al. proposed a novel image encryption algorithm that overcomes the limitations associated with one-dimensional (1D) maps and multidimensional (MD) maps. They adopted a hybrid approach, combining three modified and enhanced 1D chaotic maps, providing enhanced security. The encryption process utilizes a key image to initialize the chaotic maps. Moreover, the incorporation of ExtraParam extracted from the plaintext image adds sensitivity to bit changes during chaotic map initialization. This method comprises two core phases: confusion and diffusion, and it has proven its efficacy through rigorous testing and cryptanalysis. In^44^, Liu et al., introduced a cluster of one-dimensional quadratic chaotic maps based on topological conjugate theory. These 1D chaotic maps feature three tunable parameters, significantly expanding the parameter space compared to traditional 1D maps. The theoretical analysis validates their chaotic nature, as they are topologically conjugated with logistic chaotic maps. The paper presents an example of a 1D quadratic chaotic map and showcases several numerical simulations that confirm the ideal chaotic characteristics, aligning with the theoretical analysis. In^45^, Shraida et al. introduced a proficient technique for encrypting color images, which integrates DNA encoding and utilizes two variations of hyper-chaotic maps. The process consists of three steps: initializing conditions for generating Lorenz chaotic maps through a Secure Hash Algorithm (SHA) applied to a plain image, performing a confusion procedure using Lorenz hyper-chaotic sequences to scramble the image’s color components, and combining three approaches for diffusion: DNA encoding/decoding, addition operations between components, and XOR operations with Rossler hyper-chaotic sequences. This method ensures robust encryption for color images. In^46^, Zhang et al. introduced an image encryption scheme grounded in the Sarrus rule and the theory of linear algebra. They established the Sarrus model to scramble plain images by reordering pixel positions, producing a stereo-scrambled matrix. Additionally, the two-dimensional matrix is converted into a three-dimensional (3D) space.

### Fibonacci, DWT and DNA based image encryption schemes

Fibonacci-based image encryption schemes use the distinctive properties of the Fibonacci sequence to enhance security. However, DWT integrates multi-resolution analysis for encryption on different frequency components. While DNA-based encryption schemes add a biological element, transforming images into DNA-like structures and employing biological principles like encoding and decoding for enhanced cryptographic complexity. For instance, in^47^, Biban et al. proposed an image encryption scheme that incorporates an 8D hyperchaotic system that integrates a Fibonacci Q-Matrix (FQ-matrix). This approach enhances the security against various cyberattacks, making it suitable for real-time applications. In^48^, Zhong et al. designed a new methodology for the development of random numbers based on chaos theory. Moreover, wavelet transformations and XOR operations are also used with a chaotic matrix to enhance security. In^49^, Begum et al., proposed a hybrid blind digital image watermarking technique that integrates, Discrete Wavelet Transform (DWT), and Singular Value Decomposition (SVD). The encryption process starts with the use of the Arnold map to encrypt the watermark image. The remaining steps involve DCT, DWT, and SVD transformations applied to both the watermark and host image. In^50^, Balasamy et al. introduced a new image watermarking method that employs fuzzy-based Region of Interest (ROI) selection and wavelet transformation to secure embed encrypted watermarks securely. The process involves fuzzification of the source image for critical ROI selection, followed by a Discrete Wavelet Transform (DWT) to rearrange sub-bands using logistic mapping-derived magnitude values. This pixel swapping ensures a fully encrypted image, enhancing security and resistance to decryption. However, the challenges may arise from relying on singular values for watermark robustness and using logistic mapping for sub-band swapping, posing susceptibility to specific attacks. More detailed explanation of ROI based techniques to secure digital images can be found in^51–53^. In^54^, Chai et al. proposed a DNA-based method, involving the conversion of a plaintext image into a DNA matrix. This method employs a Chaotic Logistic Map (CLM)^55^ for the scrambling of the pixel rows and columns. After that, the sequence generated using CLM is used to create the diffusion in the image pixels. The seed parameters for this method are obtained through the application of a hashing algorithm called SHA256 on the original image. In^56^, Chen et al. incorporated a DNA coding and chaotic Henon-map to secure the digital images. Here, an S-box is initially applied to create cryptographic effects for complex DNA operations^57^. In^58^, Ganavi et al., utilized Error-Correcting Codes (ECC) with modifications to encrypt and decrypt input plaintext images. This method transforms carrier digital images into frequency bands using the Discrete Wavelet Transform (DWT) and conceals the encrypted hash of the input within high-frequency bands using the Least-Significant-Bit (LSB) technique. The approach successfully achieves data confidentiality and verifies data integrity through SHA-256. Table 1 provides an overview of the related work, encompassing both the identified limitations and the corresponding potential solutions.

**Table 1:** Summary of the related work.

Methodology	Year	Application domain	Real-world performance	Robustness against attacks	Disadvantages	Potential solutions
Chai et al.^54^	2017	DNA-based encryption	Not specified	Not specified	Lack of real-world information	Investigate practical performance
Chen et al.^56^	2020	DNA and chaotic encryption	Not specified	Not specified	Security and performance validation required	conduct real-world testing
Nematzadeh et al.^37^	2020	DNA encoding and BST	Not specified	Not specified	Further practical testing nedded	Validate and optimize for real-world use
Yadollahi et al.^38^	2020	DNA and RNA	Not specified	Not specified	Lack of real-world performance data	Investigate practical performance
Abbasi et al.^39^	2021	Chaotic evolutionary model	Not specified	Not specified	Requires performance evaluation	Conduct real- world testing
Balasamy et al.^50^	2021	Image watermarking	Secure	Resistant to decryption	Relying on SV, LM for sub-band swaping	Further Research on robust algorithms
Zhong et al.^48^	2022	Random number generation	Enhanced security	Improved security	Real-world implementation challenges	Address practical implementation issues
Begum et al.^49^	2022	Image watermarking	Security watermark	Hybrid approach	Potential for complexity in the process	Streamline the watermarking process
Benaissi et al.^28^	2023	Image encryption	Efficacious	Resilient to attack	Limited to 1D chaotic maps	Explore improved encryption
Liu et al.^44^	2023	Chaotic maps	Theoretical validation	Topological conjunction	Requires further validation	Conduct real-world testing
Shraida et al.^45^	2023	Color image encryption	Robust	Resistant to cyberattacks	Complexity in diffusion method	Optimize diffusion technique
Zhang et al.^46^	2023	Image encryption	Scrambling images	Matrix-based encryption	Limited explanation of practical use	Investigate practical applications
Biban et al.^47^	2023	Image encryption	Enhanced security	Robust against attacks	Potential for Computational overhead	Optimize for performance in real-time apps
Ganavi et al.^58^	2023	Image encryption	Data confidentiality	Data integrity	Practical verification required	Conduct practical integrity test

### Contributions of the paper

As indicated in Table 1, most current schemes exhibit weak security vulnerabilities, high computational complexity, or infeasibility for real-world implementations. Taking into account these vulnerabilities, the novelty and contributions of the proposed research are as follows:

- The application of a Fibonacci Transform (FT) for pixel scrambling is characterized by the non-linear and complex properties of the FT. These features enable the FT to intricately scramble the pixels of an image, which enhances the level of security and the encryption process.
- Implementing the TBT involves adjusting the values of the pixels by multiplying a Tribonacci array $3 \times 3$ with a vector $3 \times 1$ representing three consecutive pixels. This optimization reduces overall computational time without compromising the strength of the security measures which makes the proposed encryption scheme suitable for real-world applications.
- The proposed encryption scheme has a high-key space, which is achieved using multiple chaotic maps. The high-key space enhances the security of the digital data against brute-force attacks.
- DWT is employed to generate secondary keys that enhance the security of the propsoed encryption framework by diversifying the encryption keys.
- The nature of certain operations, such as XOR operations and DWT, allows for parallel processing that helps reduce the overall computational time.
- The proposed method employs level-2 diffusion by decomposing the diffused image into eight-bit planes and performing XOR operations with the corresponding bit planes which are extracted from the key image. This step strengthens the diffusion within the plaintext image which makes it more resistant against cryptographic attacks.

## Preliminaries

In the subsequent two subsections, we delve into the mathematical aspects of the Fibonacci Transform and the Tribonacci Transform.

### Fibonacci transform

The Fibonacci series is a sequence of numbers ($f_l =f_1, f_2, \cdots , f_n$) in which each number is the sum of the two previous numbers. It mainly starts with $f_1$ =0 and $f_2$ = 1, and the next numbers are generated by adding the previous two numbers in the sequence as shown in Eq. (1).

1 $$\begin{aligned} f_l&= \Big [f_1 =0, f_2 = 1, f_3 = \big [f_2 + f_1\big ] = 1 , f_4 = \big [f_{3} + f_{2}\big ] = 2, \\ f_5&= \big [f_{4} + f_{3}\big ] = 3, \cdots , f_n = \big [f_{n-1} + f_{n-2}\big ] \Big ] \end{aligned}$$

The generalized form of Eq. (1) is given in Eq. (2).

2 $$\begin{aligned} f_l = {\left\{ \begin{array}{ll} 0, &{}\text {if} \hspace{10pt} l=1\\ 1, &{} \text {if} \hspace{10pt} l=2 \hspace{30pt} \text {Where i} =1, 2, 3 \cdots \\ 1, &{}\text {if} \hspace{10pt} l=3\\ f_{l-1} + f_{l-2}, &{} \text {Otherwise} \end{array}\right. } \end{aligned}$$

The fibonacci and tribonacci numbers are given in Table 2 when $l \in [-7 +7]$.

**Table 2:** Fibonacci and tribonacci sequences when l = –1, $\cdots$ l = +7.

i	–7	–6	–5	–4	–3	–2	–1	0	+1	+2	+3	+4	+5	+6	+7
$f_i$	13	–8	5	–3	2	-1	1	0	1	1	2	3	5	8	13
$t_i$	1	0	1	1	2	4	7	13	24	44	81	149	274	504	927

The Fibonacci sequence, renowned for its remarkable properties, has been a subject of fascination for researchers. The researcher has employed matrix operations, such as determinants to derive class identities for generalized Fibonacci numbers [71]. The proposed research focuses on three key properties of generalized Fibonacci sequences, including, d’Ocagne’s identity (DOi), Catalan’s identity (CTi), and Cassini’s identity (CAi), and the mathematical formulation of such identities are given in Eqs. (3), (4), and (5), respectively.

3 $$\begin{aligned}&\text {DOi: }f_{i+1} \times f_{i} - f_i- \times f_{j+1} = (-1)^if_{i-j} \end{aligned}$$

4 $$\begin{aligned}&\text {CTi: }f_{i} \times f_{i} - f_{i+j} \times f_{i-j} = (-1)^{i-j}f_{i} \times f_{i} \end{aligned}$$

5 $$\begin{aligned}&\text {CAi: }f_{i+1} \times f_{i-1} - f_i \times f_i = (-1)^i \end{aligned}$$

The identities mentioned above can be represented in terms of determinants as follows:

6 $$\begin{aligned} DOi = \begin{vmatrix} f_{i+1}&f_{j+1} \\ f_i&f_{j} \end{vmatrix}, \hspace{10pt} CTi = \begin{vmatrix} f_{i}&f_{i+j} \\ f_{i-j}&f_{i} \end{vmatrix}, \hspace{10pt} CAi = \begin{vmatrix} f_{i+1}&f_i \\ f_i&f_{i-1} \end{vmatrix} \end{aligned}$$

The matrix associated with any of the mentioned identities can be employed to manipulate data in cryptography modulo *n*, but only if the greatest common divisor (gcd) of the determinant (det) and n is congruent to 1 modulo n.

- If gcd(det, n) $\equiv$ 1 mod n, the matrix can be used for data transformation in cryptography.

Considering the Fibonacci series definition, where $f_1$ = 1 and $f_2$ = 1, we can make the following observations regarding the transformation matrices:

- Regardless of the value of *i*, *CAi* can take on either +1 or -1.
- When the difference between *i* and *j* equals 1 or 2, *DOi* may assume either +1 or -1.
- If *j* belongs to the set 1, 2, *CTi* can be either +1 or -1.

According to Eqs. (3), (4), and (5), it becomes evident that Cassini’s identity can be considered a particular instance of the other two identities, as follows:

- DOi transforms into Cassini’s identity when *p* + 1 = *q*.
- CTi becomes Cassini’s identity when *p* equals 1.

### Tribonacci transform

Tribonacci transform is the extension of the fibonacci sequence and it is defined in Eq. (7).

7 $$\begin{aligned} t_{l+1} = t_l+t_{l-1} + t_{l-2}, \hspace{20pt} \end{aligned}$$

where $$t_0 = t_1 = 0$$, $$t_2 = 1$$

The negative $t_{l}$, denoted as $t_{-l}$ adheres to the recurrence relation given in Eq. (8).

8 $$\begin{aligned} t_{-l} = \begin{vmatrix} t_{l+1}&t_{l+2}\\ t_{l}&t_l+1 \end{vmatrix} \end{aligned}$$

Equations (7) and (8) yield the Tribonacci numbers, spanning values for k such as $\cdots , -7, -6$, $\cdots , +6, +7, \cdots$, as displayed in Table 2.

The encoding method utilizing the Tribonacci numbers relies on the Tribonacci numbers and involves the introduction of a matrix having three rows and three columns denoted as *M*. The matrix *M* is defined as follows:

9 $$\begin{aligned} M = \begin{bmatrix} 1&{}1&{}1\\ 1&{}0&{}0\\ 0&{}1&{}0 \end{bmatrix} = \begin{bmatrix} t_3 &{}t_{2}+t_1&{}t_2\\ t_2&{}t_1+t_0&{}t_1\\ t_1&{}t_0+t_{-1}&{}t_0 \end{bmatrix} \end{aligned}$$

With the determinant of *M* equal to 1, and the inverse of H detailed in Eq. (10).

10 $$\begin{aligned} M^{-1}&= \begin{bmatrix} 0&{}1&{}0\\ 0&{}0&{}1\\ 1&{}-1&{}-1 \end{bmatrix} \\ {}&= \begin{bmatrix} t^2_0 - t_{-1}t_1&{}t_1t_2 - t_0t_1&{}t^2_1 - t_0t_2\\ t^2_1 - t_0t_2&{}t_0t_3 - t_1 - t_2&{}t^2_2 - t_1t_3\\ t_0t_2 + t_{-1}t_2 - t^2_1 - t_0t_1&{}t^2_1 + t_1t_2 - t_0t_3 - t_{-1}t_3&{}t_1t_3 + t_0t_3 - t^2_2 - t_1t_2 \end{bmatrix} \end{aligned}$$

The calculation of the positive powers of *M*, specifically $M^l$, for *l* in the set of natural numbers, is carried out as follows:

11 $$\begin{aligned} M^l = \begin{bmatrix} l_{l+2}&{}t_{l+1} + t_l&{}t_{l+1}\\ t_{l+1}&{}t_{l} + t_{l-1}&{}t_{l}\\ t_{l}&{}t_{l-1} + t_{l-2}&{}t_{l-1} \end{bmatrix} \end{aligned}$$

The computation of the negative powers of *M*, denoted as $M^{l}$ for *l* belonging to the set of natural numbers, is detailed in Eq. (12).

12 $$\begin{aligned} M^{-1} = \begin{bmatrix} t^2_{l-1} - t_{l-2}t_l&{}t_{l-2}t_{l+1} - t_{l-1}t_l&{}t^2_l - t_{l-1}t_{l+1}\\ t^2_l - t_{l-1}t_{l+1}&{}t_{l-1}t_{l+2} - t_{l}t_{l+1}&{}t^2_{l+1} - t_{l}t_{l+1}\\ (t_{l-1} + t_{l-2})t_{l+1}&{}t_{l}(t_{l} + t_{l+1})&{}(t_{l}t_{l-1})t_{l+2}\\ (t_{l} + t_{l-1})t_{l}&{}(t_{l-1} + t_{l-2})t_{l+2}&{} (t_{l+1} + t_{l})t_{l+1} \end{bmatrix} \end{aligned}$$

Equations (11) and (12) can be readily derived through the application of mathematical induction. Utilizing the previously defined $M^z$ for *z* within the set of integers, the following properties can be readily demonstrated:

$${\textbf {Z1: }} M^p =M^{p-1} + M^{p-2} + M^{p-3}$$

$${\textbf {Z2: }} M^pM^q =M^{q}M^p = H^{p+q}(p,q \in (-\infty +\infty ))$$

$${\textbf {Z3: }} det M^p = 1$$

Hence, based on the preceding discourse, it’s evident that a Tribonacci matrix $M^{i}$, regardless of the specific index *i*, possesses the capability to convert data into an alternate domain. Moreover, it’s important to highlight that the original data can be restored, due to the invertibility of $M^i$. Consequently, $M^i$ can be effectively integrated into encryption schemes.

### Six-dimensional hyperchaotic map

**Figure 2:**
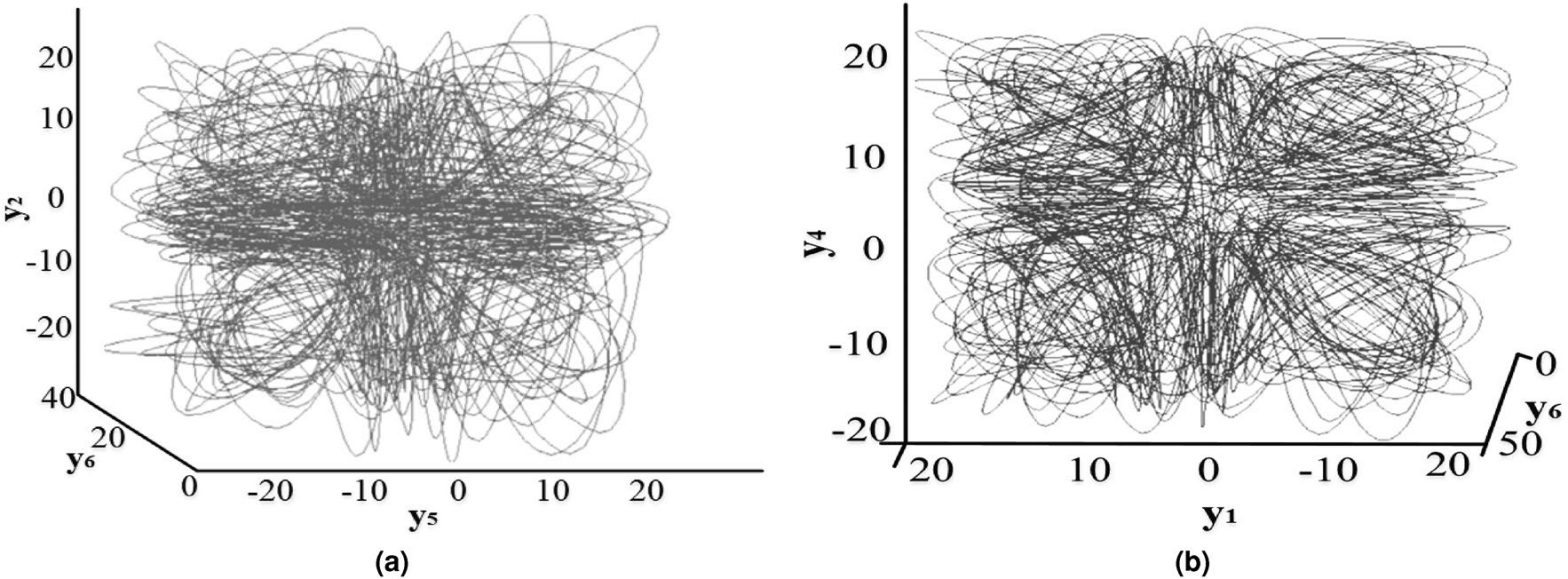
The hyperchaotic attractors of the system 13.

In^59^, Grassi et al. introduced a novel four-wing hyperchaotic attractor created through the coupling of two identical Lorenz systems, as described in Eq. (13).

13 $$\begin{aligned} {\left\{ \begin{array}{ll} \dot{y_1} = b(y_2 - y_1)\\ \dot{y_2} = cy_1 - y_2 - y_1y_3 + \eta _1(y_4 - y_5)\\ \dot{y_3} = y_1y_2 - dy_3\\ \dot{y_4} = b(y_5 - y_4)\\ \dot{y_5} = cy_4 - y_5 - y_4y_6 + \eta _2(y_1 - y_2)\\ \dot{y_6} = y_4y_5 - dy_6 \end{array}\right. } \end{aligned}$$

where the variables *b*, *c*, and *d* represent positive system parameters, while $\eta _1$ and $\eta _2$ are the coupling parameters. When specific values are assigned to these parameters, such as b = 10, c = 28, d = 8/3, and $\eta _1$ = $\eta _2$ = 0.05, the system given Eq. (13) produces the distinctive four-wing attractors depicted in Fig. 2. In the context of the subsequent cryptosystem, the parameters $b, c, \eta _1$ and $\eta _2$, and the initial conditions $y_\rho (0)$ (where $\rho \in [1, 6]$) are treated as secret keys. These secret keys, in combination with the plaintext image, are utilized to generate the key streams essential for confusion in the proposed encryption technique.

## Proposed encryption procedure

The suggested encryption procedure comprises five primary phases:

1. The creation of initial secret keys at level-1 via the utilization of a chaotic map.
2. Implementing a confusion operation employing the Fibonacci series.
3. Employing a level-1 diffusion operation with the assistance of the Tribonacci series.
4. Level-2 diffusion operation using bit-plane extraction method.
5. Incorporating of Discrete Wavelet Transform (DWT) in the proposed encryption process.
6. Implementing S-box within DWT sub-bands for level-3 diffusion.

**Figure 3:**
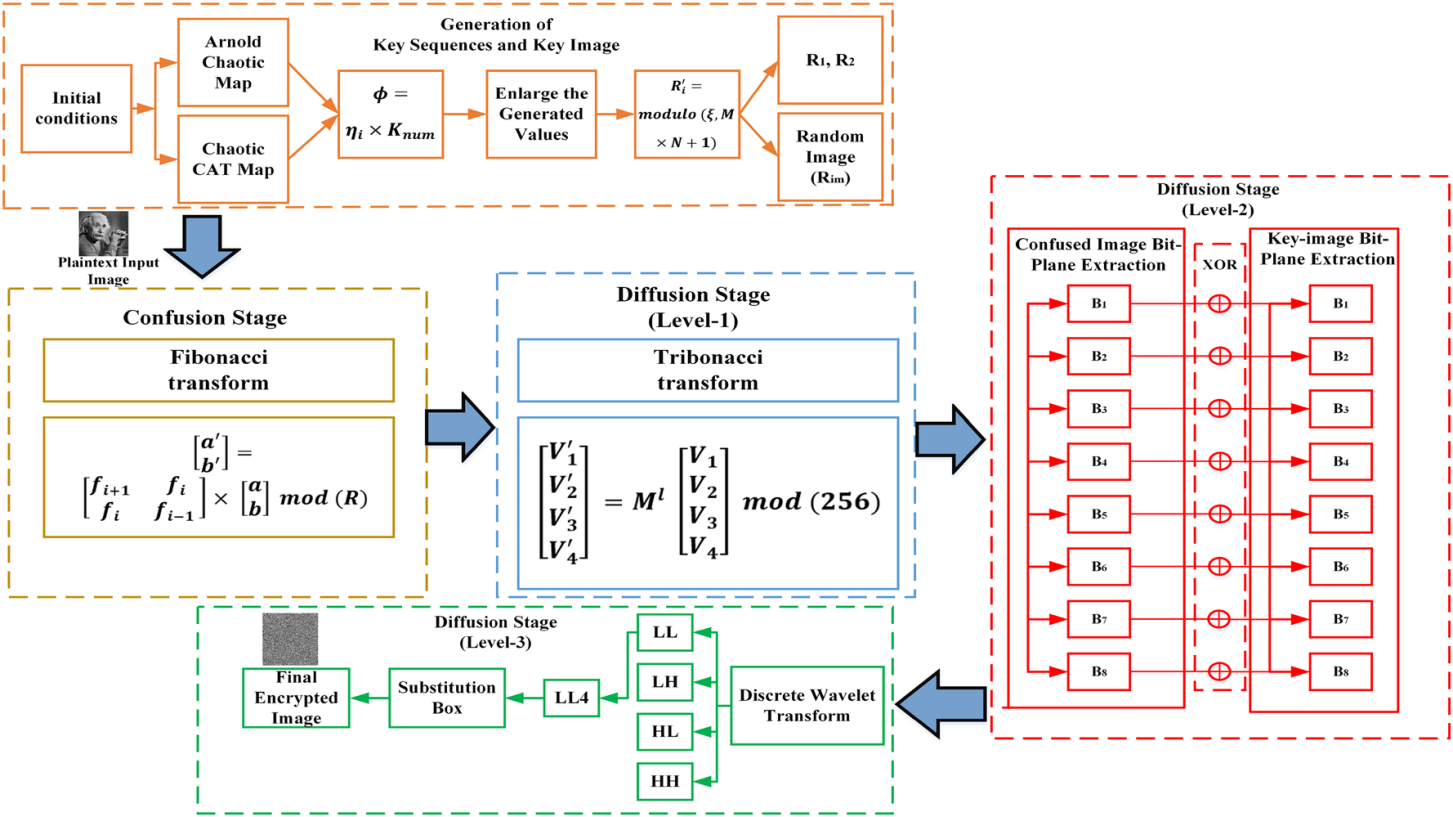
Flow of the proposed encryption scheme.

The illustration of the proposed encryption approach is given in Fig. 3. Further elaboration on the encryption process is provided in the subsequent sections.

### Random sequences and key image generation process

In the generation of the secret of level-1, multiple henon chaotic map is used. This level-1 secret key is a two-dimensional random image having no meaningful information present in it and it will be used for incorporating the exclusive OR operation to modify the intensities of the original image. Algorithm 1 provides the step-by-step details of the key generation procedure. Additionally, a 256-bit key size is employed for both encryption and decryption operations. Various initial conditions have been utilized as secret keys to produce random sequences and a key image.

**Algorithm 1:**
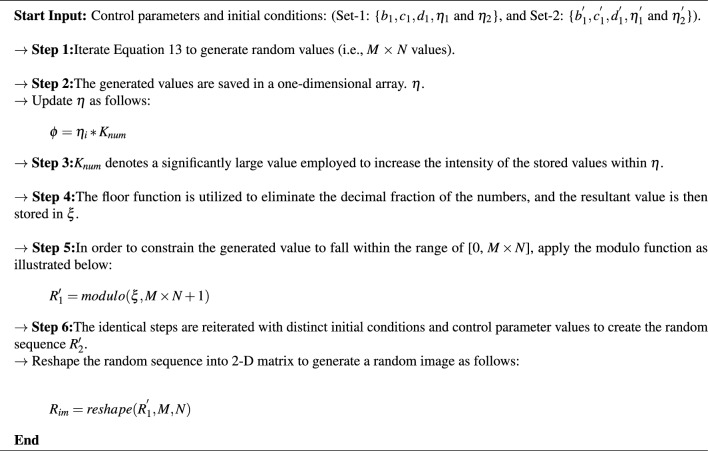
Generation of random sequences and key image $K_{im}$.

### Confusion stage

During the phase of transformation, a Fibonacci transformation is introduced to scramble the positions of the pixels. It’s important to highlight that any matrices corresponding to *CAi*, *DOi*, or *CTi* can be utilized as transformation matrices to scramble the pixels. Notably, it’s possible to parameterize these transformations using a single variable. It’s worth mentioning that Cassini’s identity is a specific case of the other two identities.

In our experimental setup, we specifically employed *CAi* during the confusion phase. The key to this confusion denoted as $k_c$, is instrumental in determining the value of *j* for *CAi*. Subsequently, this value of *i* is used to scramble the pixels. To compute *i*, we consider the formula $i = \text {mod}(k_c, P) + 3$, where *P* represents a prime number with a moderate value, and this value is made public.

For an image (*I*) with dimensions $R \times C$, where *R* and *C* show the number of rows and columns of the image pixels, and $R = C$. The pixel’s location is indicated as (*a*, *b*), and subsequent to the transformation, the new coordinates are expressed as $(a', b')$. This relationship is supported by Eq. (14).

14 $$\begin{aligned} \begin{bmatrix} a'\\ b' \end{bmatrix} = \begin{bmatrix} f_{i+1} &{} f_{i}\\ f_{i}&{} f_{i-1} \end{bmatrix} \times \begin{bmatrix} a\\ b \end{bmatrix} mod (R) \end{aligned}$$

The procedure for the confusion process is demonstrated in Algorithm 2. It is notable that, with the same key, an identical transformation can be deduced. As a result, the confusion phase exhibits invertibility.

**Algorithm 2:**
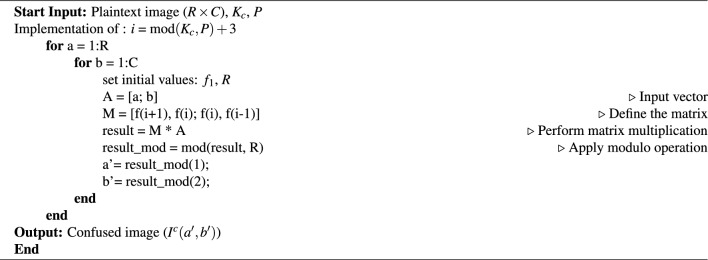
Confusion stage (Level-1).

### Diffusion stage

In order to induce level-2 confusion within $I^{c}(a^{'},b^{'})$, a pair of key sets, specifically, set-1 and set-2, are employed. Set-1 serves the purpose of conducting pixel-wise scrambling, while set-2 is applied to execute row-wise and column-wise scrambling procedures, resulting in the creation of a newly confused image denoted as $I^{'c}(a^{'},b^{'})$.

The transformation in the confusion phase retains the pixel intensities without any alteration. Consequently, the intensity profile of both the original unaltered image and the $I^c(a',b')$ image remains indistinguishable. This aspect introduces a potential security concern as an adversary might be able to make educated guesses about the original image based on the histogram profile of the scrambled image.

To address this issue, two different operations are implemented in this stage, each aimed at achieving the same objective. These operations consist of:

1. The application of XOR between the $I^c(a',b')$ and the $K_{im}$. This XOR operation is a pivotal step as it significantly alters the intensity values of the pixels within the $I^c(a',b')$ image. This XOR operation is executed at the initial stages of the diffusion process, and for the subsequent iterations in the diffusion phase, this operation is omitted.
2. The transformation of the image, which results from the initial XOR operation, is achieved through the application of the tribonacci transform, as given in Eq. (15).

15 $$\begin{aligned} \begin{bmatrix} V'_1\\ V'_2\\ V'_3\\ V'_4\\ \end{bmatrix} = M^l \times \begin{bmatrix} V_1\\ V_2\\ V_3\\ V_4\\ \end{bmatrix} \text {mod}(256) \end{aligned}$$

where $$\begin{bmatrix} V_1\\ V_2\\ V_3\\ V_4\\ \end{bmatrix}$$ shows the set of three-pixel values and $M^l$ is the transformation matrix. The inverse of the tribonacci transform will be:

16 $$\begin{aligned} \begin{bmatrix} V_1\\ V_2\\ V_3\\ V_4\\ \end{bmatrix} = M^{-l} \times \begin{bmatrix} V'_1\\ V'_2\\ V'_3\\ V'_4\\ \end{bmatrix} \text {mod}(256) \end{aligned}$$

These dual operations effectively modify the intensity values of the pixels in $I^c(a',b')$, ensuring that the pixel intensities become sufficiently scrambled. This process bolsters the security of the data by preventing any meaningful predictions about the original image based on the intensity profiles of the $I^c(a',b')$ image.

In the transformation described above, a set of four pixels is required simultaneously. Therefore, the image’s pixels are organized into groups, each comprising four pixels. Let’s assume there are *N* number of groups labeled as $N_1, N_2, \cdots , N_N$, and the pixels within each group $N_i$ are represented as $\{V_1, V_2, V_3\}$ (i.e., $N_i = \{V_1, V_2, V_3\}$). There are three possible scenarios to consider:

**Case-1:** The image contains a total of 4L pixels.

- **(a)** During the encryption process, each group $N_i$ (where $1 \le i \le L$) is subjected to transformation using Eq. (15) to yield the transformed group $N^{'}$ i, i.e., $N_i = TT(N_i)$.
- **(b)** When decrypting, the transformed group $N^{'}$ *i* (for $1 \le i \le L$) is reversed to its original form $N_i$, with the assistance of Eq. (15) (i.e., $N_i = ITT(N^{'}_i))$.

**Case-2:** The total number of pixels in the image is $3(L - 1) + 1$, which can be expressed as $N_L = \{V_L, 1\}$.

During the encryption process:

- **(a)** Initially, the first $(L - 1)$ groups ${N_1, N_2, N_3, N_{L - 1}}$ undergo transformation using Eq. (15).
- **(b)** Subsequently, $N_L$ is redefined as $N_L = \{V^{'}_{L 1, 2}, V^{'}_{L -1, 3}, V_L,1\}$ and subjected to transformation, resulting in $L^{'}_L = \{V^{''}_{L-1, 2}, L^{''}_{L, 3}, L^{'}_{L,1}\} = TT(N_L)$ using Eq. (15).
- **(c)** The resulting image with diffusion is defined as follows:
  17 $$\begin{aligned} N_1^{'}N_2^{'}|| \cdots || N_{L - 2}^{'} \{ V^{'}_{L - 1}, 1\} \end{aligned}$$

In the decryption process, the steps are as follows:

- (a) Begin by inversely transforming the first $L - 2$ groups of the diffuse image, resulting in $\{N_1, N_2, \cdot \cdot \cdot , N_{L-2}\}$, where each $N_i$ is obtained as $N_i = ITT(N^{'}_i)$ for $1 \le i \le L2$.
- (b) Following that, calculate $\{V^{'}_{L-1,2}, V^{'}_{L-1,3}, V_{L,1}\}$ by applying the inverse transformation to $N^{'}_L$, yielding $\{V^{'}_{L-1,2}$, $V^{'}_{L-1,3}$, $V_{L,1}\} = ITT(N^{'}_L)$.
- (c) Subsequently, perform the computation for ${V_{L-1, 1}, V_{L-1, 2}, V_{L-1, 3}}$ by applying the inverse transformation to the corresponding elements $\big [V^{'}_{L-1, 1}, V^{'}_{L-1, 2}, V^{'}_{L-1, 3}\big ]$.

The previous version of the diffused image can be reconstructed as $N_1|$ $|N_2||$ $\cdots ||$ $V_{L-2}||$ $\{V_{L-1,1}, V_{L-1,2}$ $, V_{L-1,3}\}$ $||\{ V_{L,1}\}$.

**Case-3:** In the case of $3(N - 1)$ + 2 pixels, which is denoted as $N_L = \{V_{L, 1}, V_{L, 2}\}$, during the encryption process:

- (a) Initially, the first $(L - 1)$ groups are transformed, resulting in $N^{'}_1, N^{'}_1, \cdots , N^{'}_{L - 1}$.
- (b) The pixel $V^{'}_{r-1,3}$ from $N^{'}_{L-1}$ is combined with Gr to form $N_L = \{V^{'}_{L-1,3}, V_{L,1}, V_{L,2}\}$.
- (c) $N_L$ is then subjected to transformation using Eq. (15), resulting in $N_L^{'} = \{V^{''}_{L-1, 3}, V^{'}_{L, 3}, V^{'}_{L, 2}\}$.
- (d) The diffuse image is formed as $N^{'}_1||N^{'}_2|| \cdots ||N^{'}_{L-2}||NL^{'}|| \{ V^{'}_{L-1,1}, V^{'}_{L-1, 2}\}$.

For the decryption process,

- (a) To begin, the first $L - 2$ groups of the diffuse image are inversely transformed, resulting in $\{N_1, N_2, \cdots , N_{L-2}\}$ (where $N_i = ITT(N^{'}_i)$ for $1 \le i \le L - 2)$.
- (b) Following that, the transformation $ITT(N^{'}_L)$ is used to compute $\{V^{'}_{L-1, 3}, N_{L,1}, N_{L,2}\}$.
- (c) Subsequently, the transformation $ITT\big [N^{'}_{L-1,1}, V^{'}_{L-1,2}, V^{'}_{L-1,3}\big ]$ is applied to compute $\{V_{L-1,1}$, $V_{L-1,2}$, $V_{L-1,3}\}$.

The image that existed prior can be reconstructed as $N_1 ||N_2|| \cdots || N_{L-2}||\{ V_{L-1,1}$, $V_{L-1,2}$, $V_{L-1,3}\}$ $||\{ V_{L,1}$, $V_{L,2}\}$.

### Bit-plane extraction

Bit-planes $(B_i)$ are the binary images that can be extracted from digital images. Mathematically, $B_i$ can be extracted using Eq. (18).

18 $$\begin{aligned} {\left\{ \begin{array}{ll} B_1 = (\frac{P(a,b)}{2^0})mod (2),\hspace{5pt} B_2 = (\frac{P(a,b)}{2^1}) mod (2)\\ B_3= (\frac{P(a,b)}{2^2})mod (2), \hspace{5pt}B_4 = (\frac{P(a,b)}{2^3})mod (2) \\ B_5 = (\frac{P(a,b)}{2^4})mod (2), \hspace{5pt} B_6 = (\frac{P(a,b)}{2^5})mod (2)\\ B_7 = (\frac{P(a,b)}{2^6})mod (2),\hspace{5pt} B_8 = (\frac{P(a,b)}{2^7})mod (2) \end{array}\right. } \end{aligned}$$

where *P*(*a*, *b*) represent the plaintext image and $B_1, B_2, \cdots , B_8$ are its binary bit-planes.

**Figure 4:**
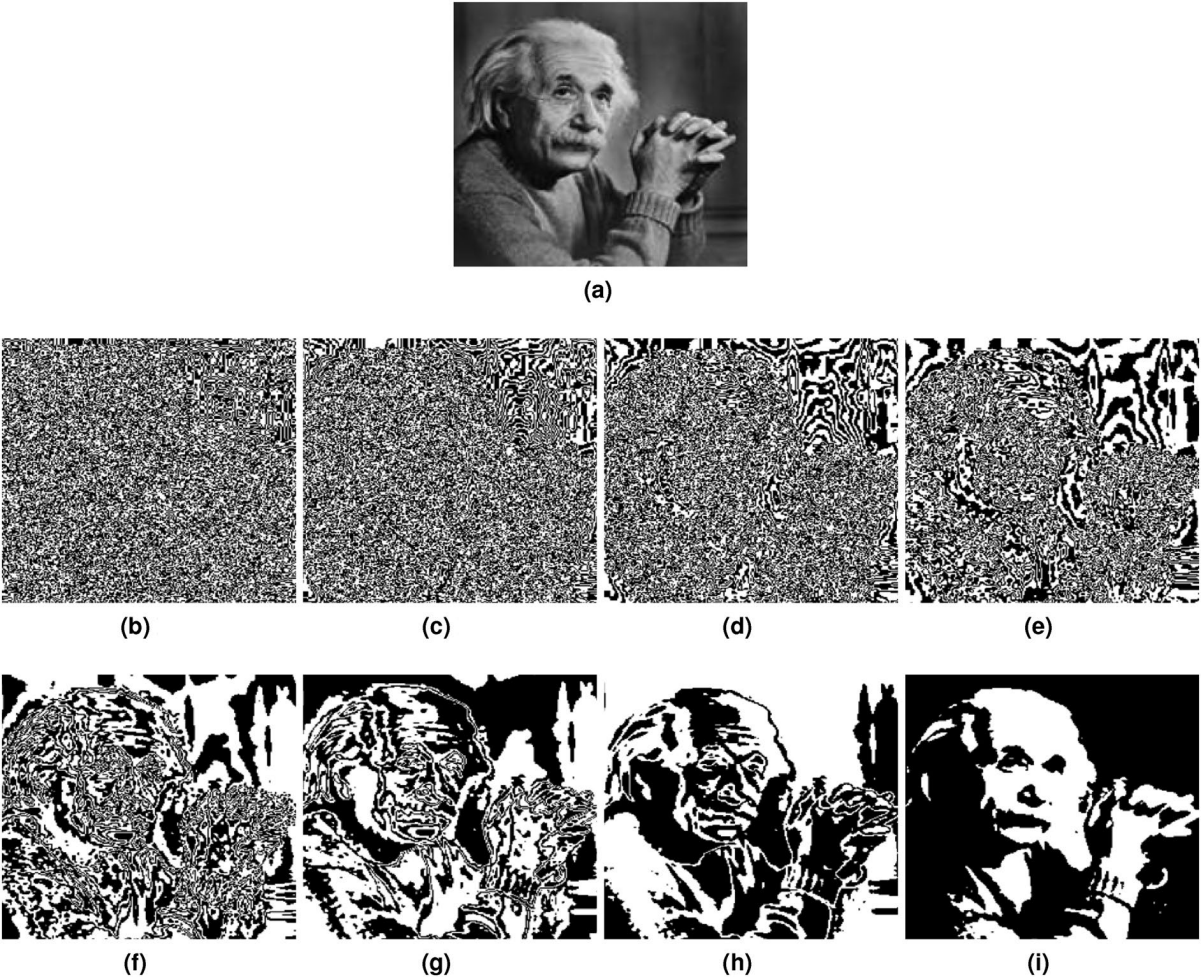
Extracted bit-planes form plaintext image. (**a**) Plaintext image. (**b**) B1. (**c**) B2. (**d**) B3. (**e**) B4. (**f**) B5. (**g**) B6. (**h**) B7. (**i**) B8.

The extracted $B_i$ can exhibit variations in the quantity of information they contain. For instance, the set of the first four bit-planes ($B_8, B_7, B_6, B_5$) has the maximum information, while the group of the second four bit-planes ($B_4, B_3, B_2, B_1$) holds the minimum information, as illustrated in Fig. 4.

The percentage content within each bit-plane can be computed using Eq. (19), and the resultant numerical values are displayed in Table 3.

19 $$\begin{aligned} P_i = \frac{2^{i-1}}{\sum _{j=1}^{8}2^{j-1}}, \hspace{40pt} \text {where } i \in [1,8] \end{aligned}$$

**Table 3:** Information percentage.

$B_i$ (i = 1, 2, $\cdots$, 8)	Percentage information
1	0.392
2	0.784
3	1.568
4	3.137
5	6.274
6	12.549
7	25.098
8	50.196

The diffused image is further encrypted using the bit-plane extraction method. The mathematical steps are given below:

To explain the mathematics behind the encryption of diffused image, a portion of the diffused image of size $3 \times 3$ is taken.

A $3 \times 3$ portion of the diffused image is:

$$\begin{aligned} D_{img}= \begin{bmatrix} 150&{}26&{}210\\ 35&{}127&{}206\\ 42&{}169&{}166 \end{bmatrix} \end{aligned}$$

The binary representation of $D_{img}$ will be:

$$\begin{aligned} D_{bin} = \begin{bmatrix} 10010110 &{}000110100&{}11010010 \\ 00100011 &{}01111111&{}11001110\\ 00101010&{}10101001&{}10100110 \end{bmatrix} \end{aligned}$$

To obtain the $B_i$ from $D_{bin}$, examine the corresponding bit of each pixel. For example, for $B_8$, select the $8^{Pth}$ bit from every pixel. Likewise, for $B_7$, select the $7^{th}$ bit from each pixel, and so forth. The resulting set of eight $B_i$ derived from $D_{bin}$ will be:

$$\begin{aligned} B_8= \begin{bmatrix} 0&{}1&{}1 \\ 1&{}0&{}0\\ 0&{}1&{}0 \end{bmatrix},B_7= \begin{bmatrix} 1&{}0&{}1 \\ 1&{}1&{}1\\ 1&{}0&{}0 \end{bmatrix},B_6= \begin{bmatrix} 1&{}0&{}1\\ 1 &{}0&{}0\\ 1&{}0&{}1 \end{bmatrix},B_5= \begin{bmatrix} 0&{}1&{}0\\ 1&{}1&{}1\\ 0&{}0&{}0 \end{bmatrix} \end{aligned}$$

$$\begin{aligned} B_4= \begin{bmatrix} 0&{}0&{}1\\ 1&{}0&{}1\\ 1&{}0&{}1 \end{bmatrix},B_3= \begin{bmatrix} 1&{}1&{}0 \\ 0&{}0&{}0\\ 1&{}0&{}0 \end{bmatrix},B_2= \begin{bmatrix} 0&{}1&{}0\\ 1&{}0&{}1\\ 1&{}1&{}0 \end{bmatrix},B_1=5 \begin{bmatrix} 0&{}0&{}0\\ 0&{}0&{}0\\ 0&{}0&{}0 \end{bmatrix} \end{aligned}$$

If the positions of the values within the $P-SI_i$ are rearranged, the resulting permuted bit-planes, denoted as $(PB_i)$, will be:

$$\begin{aligned}PB_8= \begin{bmatrix} 1&{}1&{}0\\ 0&{}0&{}0\\ 1&{}0&{}1 \end{bmatrix},PB_7= \begin{bmatrix} 0&{}1&{}1\\ 1&{}0&{}1\\ 1&{}1&{}0 \end{bmatrix},PB_6= \begin{bmatrix} 0&{}1&{}0\\ 1&{}1&{}1\\ 0&{}0&{}1 \end{bmatrix},PB_5= \begin{bmatrix} 1&{}1&{}1\\ 0&{}0&{}1\\ 0&{}0&{}0 \end{bmatrix} \end{aligned}$$

$$\begin{aligned}PB_4= \begin{bmatrix} 0&{}1&{}0\\ 0&{}0&{}1\\ 1&{}1&{}1 \end{bmatrix},PB_3= \begin{bmatrix} 1&{}0&{}1\\ 1&{}0&{}0\\ 0&{}0&{}0 \end{bmatrix},PB_2= \begin{bmatrix} 1&{}1&{}1\\ 0&{}1&{}1\\ 0&{}0&{}0 \end{bmatrix},PB_1= \begin{bmatrix} 0&{}0&{}0\\ 0&{}0&{}0\\ 0&{}0&{}0 \end{bmatrix} \end{aligned}$$

Following the permutation process, the binary values situated at position (1,1) within each $PB_i$ are consolidated to form the binary numbers composing the pixel value situated at (1,1) in the permuted image. Similarly, for acquiring the pixel (1,2) in the permuted image, the binary numbers positioned at (1,2) within each $PB_i$ are merged. This iterative process is repeated to deduce all the pixel values in the permuted image ($P_{im}$). Consequently, the resulting $P_{im}$ corresponding to $I_{BMI}$ is as follows:

$$\begin{aligned}P_{im}= \begin{bmatrix} 10010110&{}11111010&{}01010110\\ 01100100&{}00100010&{}01111010\\ 11001000&{}01001000&{}10101000 \end{bmatrix} \end{aligned}$$

After the conversion from binary to decimal, the updated matrix $p'_{im}$, denoting the transformed version of $P_{im}$, is given below.

$$\begin{aligned}P^{'}_{im}= \begin{bmatrix} 150&{}250&{}86\\ 100&{}34&{}122\\ 200&{}72&{}168 \end{bmatrix} \end{aligned}$$

From the matrix $P^{'}_{im}$, it can be seen that this is entirely different from the original matrix *I*. Likewise, the entire procedure will be reiterated for all the rows and columns.

### Discrete wavelet transform

**Figure 5:**
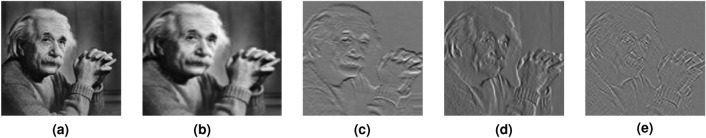
Decomposition of plaintext image into its four frequent sub-bands (When K=1). (**a**) Plaiontext image (256×256). (**b**) *LL*_1_ sub-band (128×128). (**c**) *LH*_1_ sub-band (128×128). (**d**) *HL*_1_ sub-band (128×128). (**e**) *HH*_1_ sub-band (128×128).

The wavelet transform (WT), developed in the 1980s, is a mathematical tool known for efficiently analyzing transient signals with wide frequency bands. The Wavelet Transform (WT) decomposes a signal into various representations of a mother wavelet, accomplished through shifts and scaling, enabling the segmentation of a signal into constituent wavelets. These component wavelets can then undergo further processing, including decimation, which removes some of the finer details. This process isolates the high-frequency sub-bands(HL and HH) and low-frequency sub-bands (LL and LH). The coarser details, containing the low-frequency components, are identified using larger wavelets, such as the LL-sub-band. In the context of images, the low-frequency sub-band typically contains the majority of the plaintext information, whereas the high-frequency sub-bands capture finer details, such as edges as shown in Fig. 5.

The proposed encryption technique utilizes the Haar wavelet. In this method, the Haar wavelet transform is expressed through the matrix equation $G' = W GW^T$, where *G* denotes an image with dimensions $A \times A$, *W* represents the Haar transform matrix of size $A \times A$, and $G'$ symbolizes the resultant transformed matrix of size $A \times A$, encompassing the Haar basis function $g_m(z)$. This function is defined within the interval $z \in [0, 1]$, where *m* ranges from 0 to M-1. The decomposition of this function can be comprehended as follows:

The Haar wavelet transformation is represented by a matrix equation, with $G'$ denoting the transformed image having dimensions $A \times A$, *W* representing the Haar transformation matrix of identical size, and *G* signifying the resultant transformed matrix also sized $A \times A$. This matrix encompasses the Haar basis function $g_m(z)$, defined within the interval $z \in [0, 1]$, where *m* varies from 0 to $M-1$. To elucidate this function’s breakdown, the following explanation is provided:

20 $$\begin{aligned} n = 2^r + l \end{aligned}$$

Here *r* symbolizes the highest power of 2 contained within the integer *n* while *l* represents the remainder, expressed as $l = qr - n$. Equation (21) serves to formally describe the Haar basis function.

21 $$\begin{aligned} h_c(a) = \frac{1}{\sqrt{P}}\left\{ \begin{array}{ll} 1 &{} \quad if n = 0 \hspace{8pt} \& \hspace{8pt} 0 \le a \le 1\\ 2^{r/2} &{} \quad if z> 0 \hspace{8pt} \& \hspace{8pt} l/2^r \le a<\\ {} &{}\hspace{20pt}\frac{l+0.5}{2^l}\\ -2^{r/2} &{} \quad if n > 0 \hspace{8pt} \& \hspace{8pt} (l+ 0.5)/2^r\\ &{} \hspace{20pt}\le a < \frac{l+1}{2^r}\\ 0 &{} \hspace{10pt}\text {Elsewhere} \end{array} \right. \end{aligned}$$

The matrix required to perform the two-dimensional discrete Haar wavelet transform (DHWT) can be derived by replacing the inverse transformation kernel, as given in Eq. (22).

22 $$\begin{aligned} h'(a,n) = \frac{1}{\sqrt{O}}h_n(a/O) \hspace{20pt} for \hspace{5pt} a = 0,1,2,...., M - 1 \end{aligned}$$

where *h*(*a*, *n*) will be:

23 $$\begin{aligned} h(a,n) = H' = \begin{bmatrix} h_0(\frac{0}{O}) &{} h_0(\frac{1}{O})&{}\cdots &{}h_0(\frac{O-1}{O})\\ h_1(\frac{0}{O}) &{} h_1(\frac{1}{O})&{}\cdots &{}h_1(\frac{O-1}{O})\\ h_2(\frac{0}{O}) &{} h_2(\frac{1}{O})&{}\cdots &{}h_2(\frac{O-1}{O})\\ \vdots &{}\vdots &{}\ddots &{}\vdots \\ h_{O-1}(\frac{0}{O}) &{} h_{O-1}(\frac{1}{O})&{}\cdots &{}h_{O-1}(\frac{O-1}{O})\\ \end{bmatrix} \end{aligned}$$

In 2-D digital image processing, individual rows of the image undergo a dual filter procedure that involves both a low-pass filter and a high-pass filter. Following this filtering, the outputs are subsequently downsampled by a factor of two, which leads to the formation of two distinctive information sub-bands: $L_f$ representing the approximate information sub-band, and $H_f$ representing the fine detail information sub-band, both in the horizontal direction. This entire operation is then replicated for every column of these newly generated images, resulting in the creation of four distinct frequency sub-bands.

**Figure 6:**
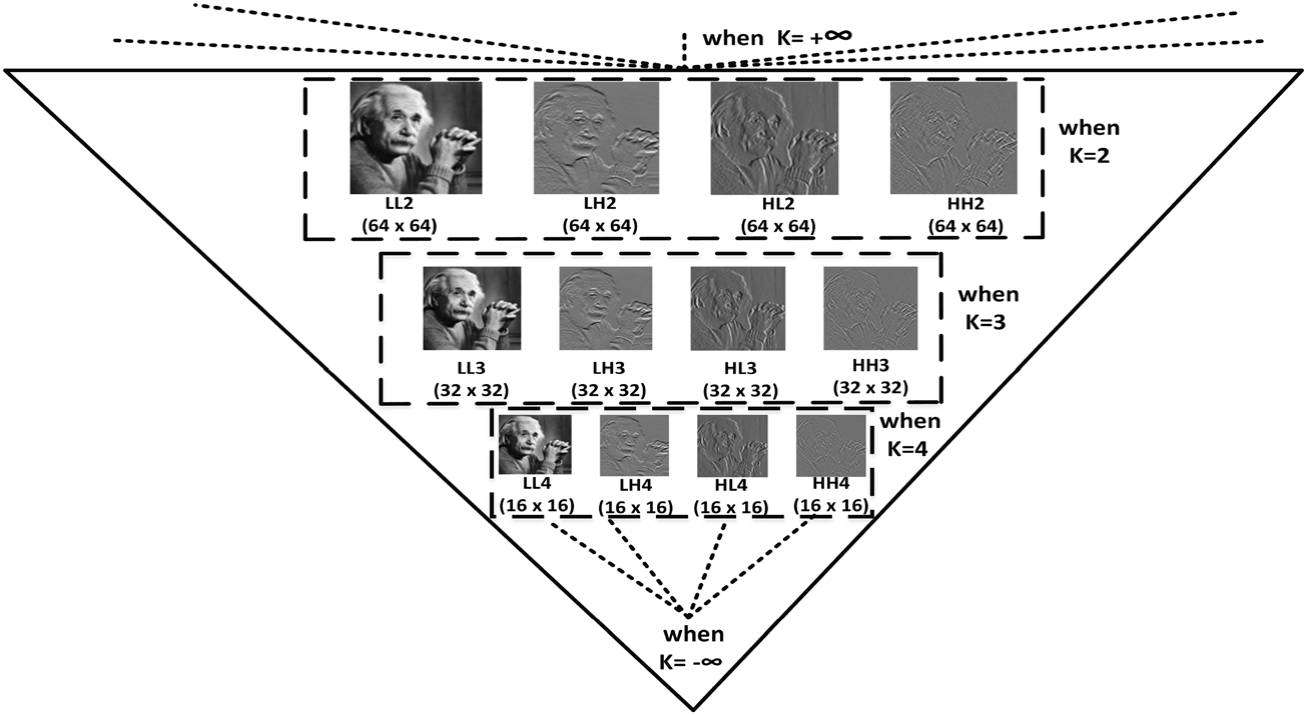
Pyramid behavior: DWT decomposition when the value of K gradually decreases from +$\infty$ to -$\infty$.

Upon subjecting the $LL_1$ sub-band to another round of 2D-DWT, four additional sub-bands are obtained: $LL_2$, $LH_2$, $HL_2$, and $HH_2$. This iterative process can be repeated up to *T* times, yielding a sequence of sub-images: $LL_T$, $LH_T$, $HL_T$, and $HH_T$. The value of *T* plays a significant role, as it determines the reduction in size of each sub-band by a factor of $2^n$, where *n* ranges from 1 to $N-1$, with *n* belonging to the interval [1, N-1]. Within the framework of the proposed approach, a value of *K* is designated as 3, meaning that with $K=4$, each sub-band’s dimensions are scaled down to $32 \times 32$ when the original image dimension is $256 \times 256$. Figure 6 illustrates a pyramid pattern of the DWT decomposition, covering level-2 (i.e. K=2) to level-4 (i.e. K=4).

This is performed to reduce the computational time of the encryption process. For example, to create diffusion in an image using a substitution box (S-box), a time of approximately 20 seconds will be taken to encrypt the image of size $256 \times 256$^60^. Therefore, creating diffusion using an S-box in a small frequency band having dimensions of $32 \times 32$ will take very little time compared to the time taken to create diffusion using an S-box in an image of size $256 \times 256$. The image $P_{im}$ is generated in the preceding step is decomposed into its four frequency sub-bands, and then only the *LL* sub-band undergoes further decomposition until its size becomes $32 \times 32$ (i.e $LL_4$). The $LL_4$ is subsequently subjected to S-box substitution, as given in^61^, to create the final encrypted image. The steps to perform the substitution in $LL_4$ are given in Algorithm 3.

The image denoted as $P_{im}$, which is produced in the previous stage, is subjected to decomposition into its four frequency sub-bands. Among these sub-bands, only the $LL sub-band$ is further decomposed until its dimensions reach a size of $32 \times 32$, referred to as $LL_4$. Following this, the $LL_4$ sub-band is then subjected to multiple S-box substitutions using the S-boxes i.e. S-box-1, S-box-2, and S-box-3 given in^61,62^, and^63^, respectively, to produce the final encrypted image ($E_f$). The specific steps for performing this substitution on $LL_4$ are given in Algorithm 3.

**Algorithm 3:**
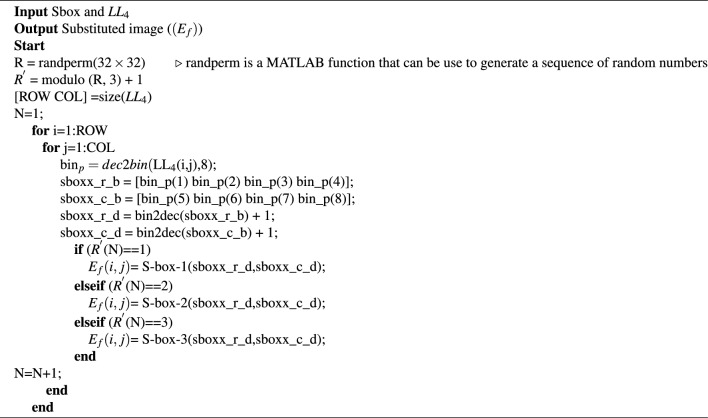
Substitution process on $LL_4$.

**Figure 7:**
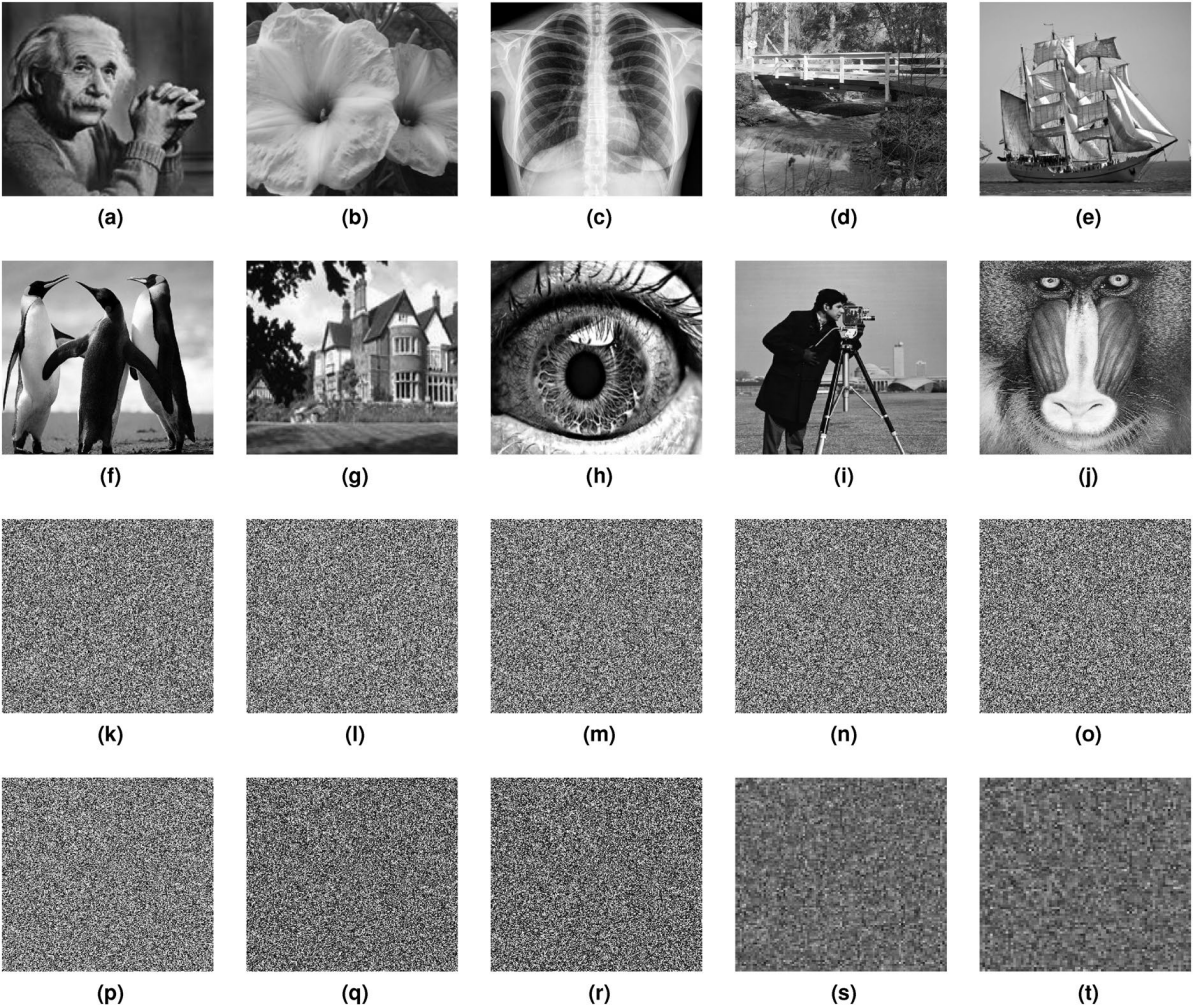
Visual encryption results: (**a**–**j**) input plaintext images, (**k**–**t**) corresponding ciphertext images.

The test images randomly sourced from Google of different sizes and the corresponding ciphertext images, which are produced using the proposed encryption method, are shown in Fig. 7. The size of each image displayed in Fig. 7a–g is $256 \times 256$. In contrast, the dimensions of each image given in Fig. 7h–j are $512 \times 512$. Upon examination of the figure, it becomes evident that there is no original plaintext image information is visible in the ciphertext images. This observation shows the efficacy of the proposed encryption scheme in successfully concealing the plaintext information.

## Experimental results and analysis

To assess the effectiveness of the proposed encryption scheme, various statistical security analyses, including entropy, contrast, correlation, contrast, mean square error, and histogram analysis, are conducted. In addition to these statistical analyses, a series of attacks, such as cropping attacks, brute force attack, and noise addition attacks, are carried out to evaluate the robustness of the proposed encryption.

### Computational time analysis

To optimize the encryption framework for real-time applications, it is necessary to minimize the computational time. This research conducts computational analyses on images of different sizes i.e. $256 \times 256$ and $512 \times 512$. Additionally, apart from assessing the computational time for encrypting images, the analysis extends to the key generation process, and decryption process. Although the key generation process is distinct from encryption, it plays a crucial role as the generated keys are utilized within the proposed encryption framework. The computational time for the propsoed encryption and decryption processes, and generating keys is measured using the built-in MATLAB command “tic toc.” Table 4 presents the statistical values calculated using the MATLAB built-in command known as ”tic toc”. Table 4 reveals that the proposed encryption framework can encrypt images of sizes $256 \times 256$ and $512 \times 512$ in less than one second. This indicates the suitability of the proposed encryption framework for real-time applications. Moreover, it is evident that the execution times for encryption and decryption processes are approximately equal. This consistency arises because the decryption process involves the same number of mathematical steps as encryption, but in reverse order, along with the inversion of each step.

**Table 4:** Computational complexity analysis (sec).

Key generation process	Size	Images	Encryption process	Decryption process
0.0001	$256 \times 256$	Einstein	0.0020	0.0021
		Flowers	0.0021	0.0022
		Tree	0.0020	0.0021
-	$512 \times 512$	Cameraman	0.0040	0.0042
		Eye	0.0041	0.0043
		Baboon	0.0040	0.0042

### Histogram Analysis

In image processing, a histogram represents the distribution of the pixel values within an image. In the case of a robust encryption scheme, the histogram of the ciphertext image should exhibit characteristics such as flatness,

In the context of a resilient encryption scheme, it is desirable that the histogram of the enciphered image shows features that include uniformity, even distribution, and a notable dissimilarity from the histogram of the original image.

**Figure 8:**
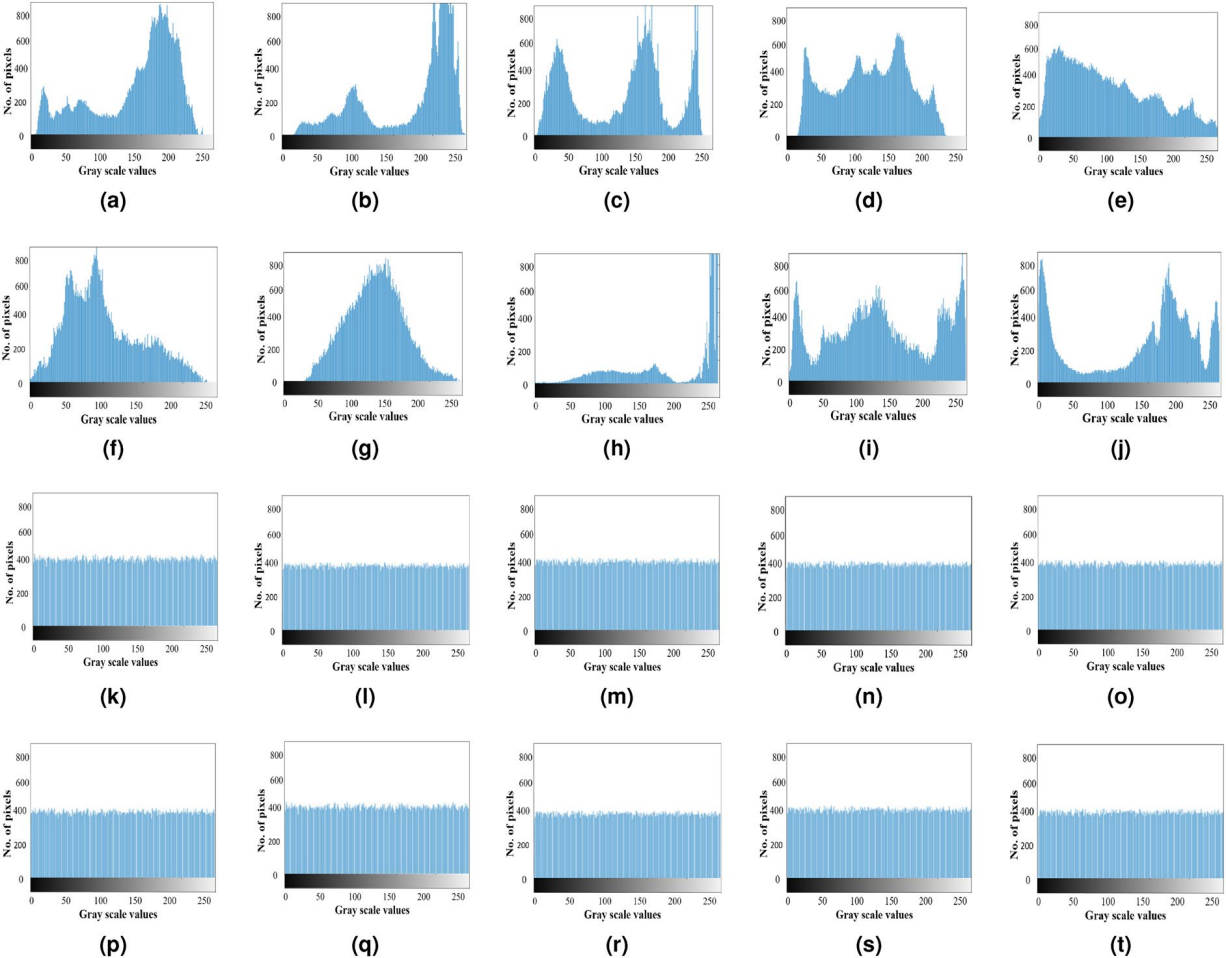
Histogram analysis: (**a**–**j**) histogram of an input plaintext images, (**k**–**t**) histogram of corresponding ciphertext images.

Figure 8 displays different histograms, illustrating that the pixel distribution within the histogram of plaintext images is relatively uniform. Furthermore, the consistency in pixel distribution signifies the encryption scheme’s capability to withstand potential histogram-based attacks.

### Histogram variance analysis

Variance is used to assess the uniformity of the pixel distribution in an image. This metric is often deemed more reliable because it provides statistical values rather than relying on histogram visualizations. This metric is typically considered more reliable because it offers statistical values instead of relying on histogram visualizations. Mathematically, this metric can be calculated using Eq. (24).

24 $$\begin{aligned} Var(P) = \frac{1}{256} \sum _{L=1}^{256}[p_i - E(P)]^2 \end{aligned}$$

In the equation, *P* represents the pixel stream, where *P* = { $p_1$, $p_2$, $p_3$ $\cdots , p_{256}$, and $p_i$ denotes the pixel value at the $L^{th}$ position. The term *E*(*P*) is computed as *E*(*P*) = $\frac{1}{256}$ $\sum _{L=1}^{256}$ $p_i$. In the context of robust encryption, low variance values are desirable.

Table 5 presents a range of variance values for the enciphered images produced by both the proposed and the encryption schemes presented in the past recent years. These variance values highlight the superior performance of the proposed scheme in comparison to the existing ones.

**Table 5:** Histogram variance analysis.

Size	Images	^64^	^65^	^66^	^67^	^68^	Proposed
$256 \times 256$	Einstein	272.371	273.379	265.630	273.136	277.3694	260.637
	Flowers	267.697	266.378	275.672	279.994	268.978	261.300
	Tree	274.370	275.336	270.039	274.698	272.451	260.633
	Man	275.637	276.336	279.987	274.831	275.379	259.596
	Boat	277.687	274.678	279.689	274.678	274.678	262.116
$512 \times 512$	Cameraman	271.678	275.298	279.164	275.245	276.315	261.378
	Eye	271.335	271.189	275.269	277.591	271.679	264.501
	Baboon	273.164	275.941	276.972	271.39	270.113	266.677

### Maximum Deviation

The performance of a cryptographic algorithm can be evaluated by measuring the deviation between the pixel values of the original and enciphered images^69^. If the deviation in pixel intensities between the original and enciphered images is maximized, it indicates a higher level of security for the encryption technique. Mathematically, the maximum deviation can be expressed as

25 $$\begin{aligned} M_A =\frac{A_0 + A_{N-1}}{2} + \sum _{L=1}^{N-2}A_L \end{aligned}$$

Here, *N* stands for the number of gray levels, and $A_L$ represents the amplitude of the histogram at the $L^{th}$ index. A higher value of $M_A$ indicates a more significant difference between the ciphertext and the original image. Table 6 presents the results of $M_A$ for the proposed scheme and the existing algorithms. The comparison reveals that the proposed encryption technique outperforms the existing schemes. However, images with high texture can yield a higher maximum deviation. Table 6 indicates a slightly higher value of the Maximum Absolute (MA) deviation compared to the proposed work. This discrepancy is attributed to the high texture present in the plaintext image employed by the authors of the existing work.

**Table 6:** Maximum deviation analysis.

Size	Images	^64^	^65^	^66^	^67^	^68^	Proposed
$256 \times 256$	Einstein	25121	25881	24985	24643	25329	25989
	Flowers	25989	25787	24692	24345	25719	26793
	Tree	26180	26047	24673	24336	25014	26249
	Man	25964	26646	24370	25042	24890	26796
	Boat	25825	25144	25012	25008	25121	25598
$512 \times 512$	Cameraman	25935	25763	25899	25864	25836	26531
	Eye	25836	25899	25968	25710	25967	26318
	Baboon	25861	25734	25931	25793	25799	26634

### Entropy

Entropy is used to assess the level of robustness in both plaintext and ciphertext images. A higher level of randomness within an image corresponds to a higher entropy value, as illustrated in Eq. (26).

26 $$\begin{aligned} Entropy \propto randomness \end{aligned}$$

The entropy can be calculated using Eq. (27).

27 $$\begin{aligned} Entropy = -\sum p(k_i) \log _2 \rho (k_i) \end{aligned}$$

where $\rho k_i$ represents the probability of occurrence for variable $i$.

The ideal entropy value is dependent on the number of bits used to represent the image. For example, in a binary image, the ideal entropy value can be 2. In the case of eight-bit images used in the proposed work, the ideal entropy value is 8.

All images utilized in the experimental results and analysis are 8-bit images. Therefore, the ideal value for each encrypted image should approach or be approximately equal to 8. Table 7 indicates that the entropy values associated with the proposed encryption scheme exhibit a significant proximity to the value of 8. Furthermore, the existing encryption scheme also attains entropy values near eight, though these values remain slightly lower than those achieved by the proposed encryption scheme.

**Table 7:** Entropy analysis.

Size	Images	^64^	^65^	^66^	^67^	^68^	Proposed
$256 \times 256$	Einstein	7.9876	7.9960	7.9936	7.9942	7.9986	7.9992
	Flowers	7.9777	7.9971	7.9936	7.9989	7.9990	7.9993
	Tree	7.9789	7.9982	7.9936	7.9951	7.9973	7.9991
	Man	7.9888	7.9940	7.9946	7.9973	7.9987	7.9990
	Boat	7.9879	7.9983	7.9982	7.9990	7.9983	7.9992
$512 \times 512$	Cameraman	7.9875	7.9961	7.9973	7.9989	7.9980	7.9994
	Eye	7.9851	7.9967	7.9941	7.9971	7.9973	7.9994
	Baboon	7.9869	7.9967	7.9982	7.9966	7.9988	7.9995

### Correlation

The correlation among the pixel values of an image reflects the degree of the intensity relationship between them. It also quantifies how similar or dissimilar pixel values are. Greater differences between pixel values indicate lower correlation^70^. This correlation relationship can be expressed mathematically as follows:

$$\begin{aligned}Correlation \propto \frac{1}{\text {pixel divergence}}\end{aligned}$$

The mathematical formula for determining the correlation among image pixels can be expressed as:

$$\begin{aligned}CorrCoff= & {} \frac{Cov(w,t)}{\sigma _w\sigma _t}, \hspace{15pt} \sigma _w = \sqrt{VAR_w}, \hspace{15pt} \sigma _t = \sqrt{VAR_t}\\ VAR (n)= & {} \frac{1}{R} \sum _{u=1}^R(n_s - E(n))^2, \hspace{15pt} Cov(n, m) = \frac{1}{R} \sum _{u=1}^R(n_s - E(n) (h_s - E(m)) \end{aligned}$$

where *E* represents the expected value operator, and $\sigma$ signifies the standard deviation.

In a plaintext image, there is typically a high correlation between pixel values because the image content is readily visible. Conversely, in a ciphertext image where pixel content is concealed, there should be a lower correlation among the pixels. Thus, it is essential that the correlation values between pixels in ciphertext images are minimized to prevent the visualization of any content in the encrypted image^71^.

Table 8 presents a comparative analysis of correlation values across different encryption schemes, including the proposed encryption technique. The data presented in Table 8 indicates that the correlation values produced by the proposed scheme are less than those observed in the existing schemes.

**Table 8:** Correlation analysis.

Size	Images	^64^	^65^	^66^	^67^	^68^	Proposed
$256 \times 256$	Einstein	0.0026	0.0016	0.0017	0.0025	0.0018	0.0001
	Flowers	0.0016	0.0014	-0.0025	-0.0019	0.0010	-0.0015
	Tree	0.0022	-0.0020	-0.0012	0.0020	-0.0014	-0.0001
	Man	0.0027	-0.0019	-0.0016	-0.0013	0.0031	-0.0015
	Boat	0.0022	-0.0079	-0.0019	0.0038	0.0049	-0.0020
$512 \times 512$	Cameraman	0.0026	-0.0036	0.0054	0.0011	0.00013	-0.0003
	Eye	0.0062	0.0012	-0.0011	-0.0032	0.001	0.0003
	Baboon	0.0046	0.0045	0.0033	0.0041	0.0034	0.0001

**Figure 9:**
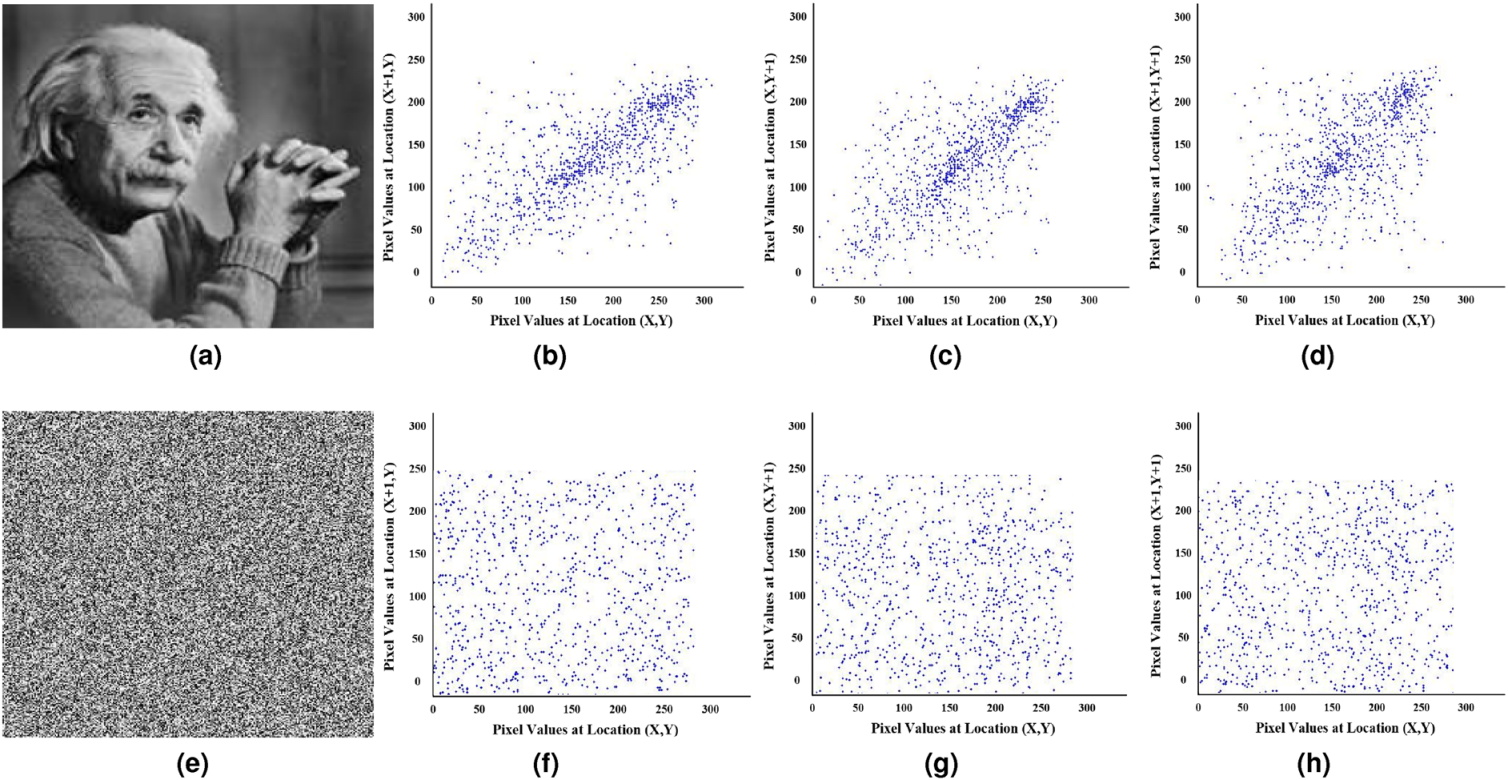
Pixel correlation analysis in horizontal, vertical, and diagonal directions. (**a**) Plaintext image. (**b**) Horizontal correlation. (**c**) Vertical correlation. (**d**) Diagonal correlation. (**e**) Ciphertext image. (**f**) Horizontal correlation. (**g**) Vertical correlation. (**h**) Diagonal correlation.

In addition to the statistical analysis of correlation, a visual assessment can be conducted using scatter plots. Figure 9(a), and 9(e) show the plaintext and ciphertext images, respectively. Whereas, Fig. 9b–d,f–h illustrate scatter diagrams for both plaintext and ciphertext images. Upon examination of these scatter diagrams, it can be seen that in Fig. 9b–d, the blue dots are closely grouped together, indicating a high pixel correlation. Conversely, in Fig. 9f–h, the blue dots are dispersed, signifying a substantial reduction in pixel correlation. This demonstrates the effectiveness of the proposed encryption scheme in mitigating pixel correlations within the images.

### Noise and clipping attack analysis

**Figure 10:**
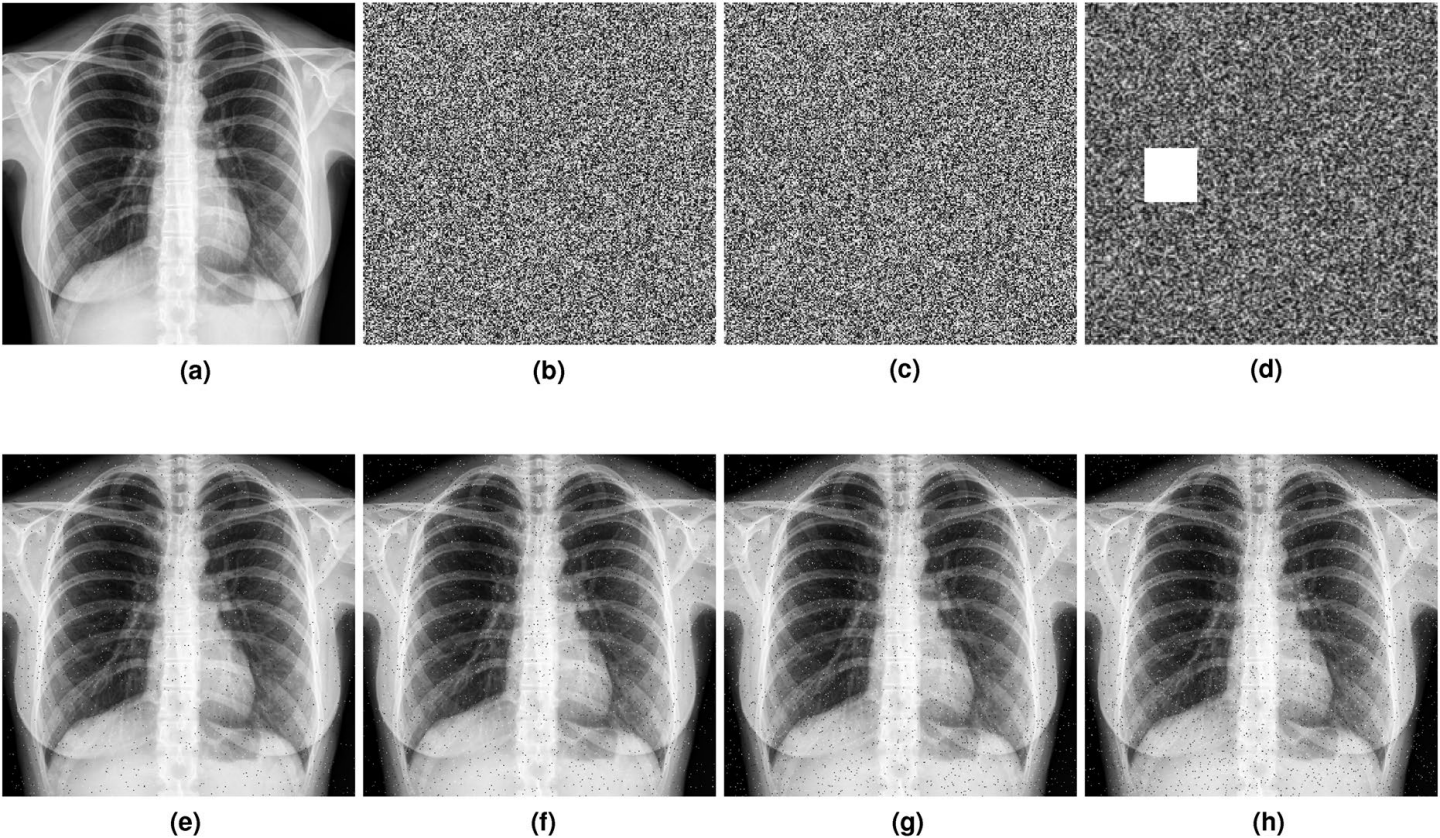
Noise and clipping attack analysis. (**a**) Plaintext image. (**b**) Encrypted image. (**c**) Noisy version of the encrypted image. (**d**) Clipped version of the encrypted image. (**e**) Decryption from the noisy encrypted image using the proposed framework. (**f**) Decryption from the clipped encrypted image using the proposed framework. (**g**) [Decryption from the noisy encrypted image using the encryption framework proposed in 72. (**h**) Decryption from the clipped encrypted image using the encryption framework proposed in 72.

To make the decryption fail, attackers commonly tamper with encrypted images by incorporating noise. An effective encryption scheme must demonstrate noise resilience to effectively mitigate noise attacks. In assessing the influence of the noise attack, 0.02 means 2% of the pixels are affected by the salt and pepper noise introduced into the encrypted image. The modified encrypted image, now impacted by salt and pepper noise, is decrypted using the proposed decryption algorithm. Figure 10a–d displays the plaintext image, the encrypted image, a noisy version of the plaintext image, and a clipped version of the encrypted image, respectively. The decryption outcomes are depicted in Fig. 10e,f, where it can be seen that although the exact pixel values are not restored, the content of the plaintext can be clearly visualized.

In contrast, attackers also attempt to compromise decryption integrity by clipping parts of the encrypted images. To evaluate the resistance of the proposed encryption framework against clipping attacks, $\frac{1}{16}$, or approximately 16.6% of the ciphertext image, is ciphered from the encrypted images. These clipped versions are then decrypted to recover the plaintext images. The decryption results from these noisy and clipped encrypted images are illustrated in Fig. 10e,f, respectively, which demonstrate that most of the content of the plaintext image remains visible in the decrypted images. Moreover, Fig. 10g,h are recovered images from noisy and cllpied encrypted images, respectively, using the encryption techniques presented in^72^. The comparison utilizes the same plaintext image, specifically a chest X-ray image, which is also used in the study presented in^72^. This comparison reveals that our proposed encryption framework is more capable of reconstructing plaintext information from both noisy and clipped versions of the encrypted images. Apart from the visual results, statistical analyses are also conducted for both noise and clipping attack analysis. The statistics are detailed in Table 9, which illustrates that the proposed method shows a slight improvement over the encryption scheme introduced in^72^.

**Table 9:** Percentage recovery form the noisy and clipped versions of the ciphertext images.

Salt & pepper noise	Clipping area	Recover a percentage from the noisy version of the encryption using the proposed encryption framework	Recover a percentage from the clipped version of the encryption using the proposed encryption framework	Recover a percentage from the noisy version of the encryption using the encryption framework proposed in^72^	Recover a percentage from the clipped version of the encryption using the encryption framework proposed in^72^
2%	$\Big (\frac{1}{6}\Big )^{th}$	97.8%	96.8%	97.4%	96.3%

## Uniqueness and the advantages of the proposed encryption framework

In this section, various features and advantages of the proposed encryption framework are provided in the domain of secure data protection, as given below:

- *Innovative techniques:* The proposed encryption framework distinguishes itself from existing encryption schemes through the strategic integration of innovative techniques. These methods are carefully chosen to concurrently enhance both security and computational efficiency.
- *Key components:* The incorporation of chaotic maps, FT, TT, and Discrete Wavelet Transform (DWT) diffusion represents a unique amalgamation. This combination contributes significantly to fortifying the robustness against cyberattacks.
- *Dynamic pixel scrambling:* The incorporation of chaotic maps and FT ensures the dynamic behavior of initial pixel scrambling.
- *Multilayered security architecture:* The introduction of TT for level-1 diffusion, along with a level-2 diffusion in which XOR operations are involved results in a multilayered security architecture.
- *Secondary key generation:* The utilization of DWT for secondary key generation and high-frequency sub-band substitution further enhances the encryption process.
- *Statistical validation:* Impressive statistical values of security parameters consistently demonstrate the effectiveness of the proposed encryption framework compared to existing state-of-the-art methods.
- *Real-world applicability:* The proposed encryption framework is suitable for real-world applications where less processing time is a critical requirement.

## Conclusion and future work

The proposed work presents a new image encryption technique that relies on several key components, including the Fibonacci transformation, chaotic maps, the DWT, bit-plane extraction, and Tribonacci transformations. To secure the image, a $2 \times 2$ Fibonacci matrix is employed for scrambling, while a $3 \times 3$ Tribonacci matrix is utilized in the diffusion phase to modify pixel values. Chaotic maps are employed to generate key sequences, which are used to introduce confusion to the image pixels. The process begins with comprehensive pixel scrambling, encompassing both row- and column-wise transformations. Therefore, the diffusion phase is initiated, which is started with the application of the TT to achieve level-1 diffusion in the scrambled image. The diffused image undergoes a level-2 diffusion through the incorporation of the bit-plane extraction technique. This involves extracting bit planes from the level-1 diffused image and a 2-dimensional key image. For a more extensive level-3 diffusion, the DWT is utilized to decompose the diffused image into four frequency sub-bands. Among these, only the $LL_1$ is substituted using the S-box to reduce computational time during encryption. Several statistical analyses are conducted to evaluate the proposed encryption technique, demonstrating its superior performance in terms of both security and computational complexity compared to existing methods.

Future work may explore the application of this encryption technique in various domains, such as securing multimedia data in cloud storage or real-time image transmission. Additionally, we could focus on optimizing the computational efficiency of the color image encryption process, which will make it even more suitable for real-time applications. Investigating the potential integration of additional advanced encryption algorithms with traditional encryption techniques can enhance overall security.

## Data Availability

The datasets used and/or analysed during the current study available from the corresponding author on reasonable request.
